# Bilateral Well Leg Compartment Syndrome Associated with Lithotomy (Lloyd Davies) Position During Gastrointestinal Surgery: A Case Report and Review of Literature

**Published:** 2009-10-14

**Authors:** Kuen Yeow Chin, Sarah Jane Hemington-Gorse, Catharine Mary Darcy

**Affiliations:** Welsh Centre for Burns and Plastic Surgery, Morriston Hospital, Swansea SA6 6NL, United Kingdom

## Abstract

**Introduction:** Well leg compartment syndrome is a rare complication that can occur after prolonged surgery in lithotomy position. We stress the importance to recognize high-risk patients for this complication and finding ways to reduce this risk. We also emphasize high level of suspicion of at risk patients and early recognition of signs and symptoms to enable early diagnosis and treatment. **Methods:** Presentation of a case report of bilateral well leg compartment syndrome with review of literature and discussion of the pathophysiology, risk factors and treatment of this condition. **Result:** This is a possible complication of surgery associated with significant morbidity and mortality. Published papers have suggested possible ways to reduce this risk and achieve early diagnosis. **Conclusion:** Clinicians should be aware of the aware of the risk factors for developing well leg compartment syndrome and in patients and have high index of suspicion when assessing them. Further studies looking at the risks, benefits and feasibility of suggestions on ways to reduce this risk is required.

Compartment syndrome of the lower leg occurs when increased osteofascial compartmental pressure leads to decreased tissue perfusion to the leg. It can develop after prolonged surgery in the lithotomy (Lloyd Davies) position. When a patient is in this position, lower leg compartment systolic pressure falls. If this decrease in systolic blood pressure falls below the perfusion pressure, tissue ischemia occurs.^1^ This effect is increased by the Trendelenburg position (putting the patient in a head-tilt position). After a period of tissue ischemia when circulation returns to normal (ie, postsurgery), reperfusion injury occurs, leading to capillary leakage and tissue edema that form a vicious cycle that can lead to the development of compartment syndrome.^2^ Signs and symptoms include paresthesia, pain out of proportion to the injury, pain on passive stretch, tight calf, and, in more advanced stage, absent peripheral pulses.^3^ High creatine kinase levels also make the diagnosis likely.^3^ The management of compartment syndrome is immediate fasciotomies to release all 4 leg compartments. Compartment syndrome can lead to significant morbidity and mortality. Muscle and nerve damage, contractures, limb amputation, renal failure, and death are among the complications of leg compartment syndrome.

## CASE REPORT

The patient was a 44-year-old woman with ulcerative colitis. She was admitted for proctectomy, pouch formation, and ileostomy. The patient was a nonsmoker and led an active lifestyle. She had a body mass index of 26.6.

The surgery was carried out under general anesthesia, with thoracic epidural analgesia. The patient had antiembolic stockings throughout the procedure without intermittent pneumatic compression. She was placed in a lithotomy (Lloyd Davies) position for the procedure with Lloyd Davies stirrups and no head-down tilt (lithotomy-Trendelenburg position). The patient was maintained in this position throughout the procedure. The procedure lasted 7 hours with 980 mL of blood loss during the procedure, and the patient's arterial blood pressure was maintained at 90/55 mmHg or above throughout the procedure.

In the recovery unit, the patient complained of severe cramping-type pain in both legs but there were no swelling or tenderness to palpation. The pain was initially attributed to the surgery and the patient's analgesics were increased. Her epidural analgesia continued for 2 days before it was changed to patient controlled analgesia morphine infusion. Two days later, on examination, patient's left leg was swollen, tender, and hard. Posterior tibial and dorsalis pedis pulses were present in both legs. Doppler ultrasound scan carried out on the left leg showed thrombosis of left popliteal and calf veins. Patient was infused with heparin. On evening of day 2 postoperative, the patient's left leg was found to be cold and mottled with her calf swollen and tender. There was paresthesia in her left leg and foot. Left dorsalis pedis and posterior tibial pulses were absent but present in her right leg. Plasma creatine kinase levels was found to be 35,000, but the renal function remained within normal limits. Although compartment pressures in both legs were not measured, a clinical diagnosis of compartment syndrome was made. Emergency bilateral fasciotomies were carried out with 2 incisions to release all 4 compartments in both legs.

**Figure 1:**
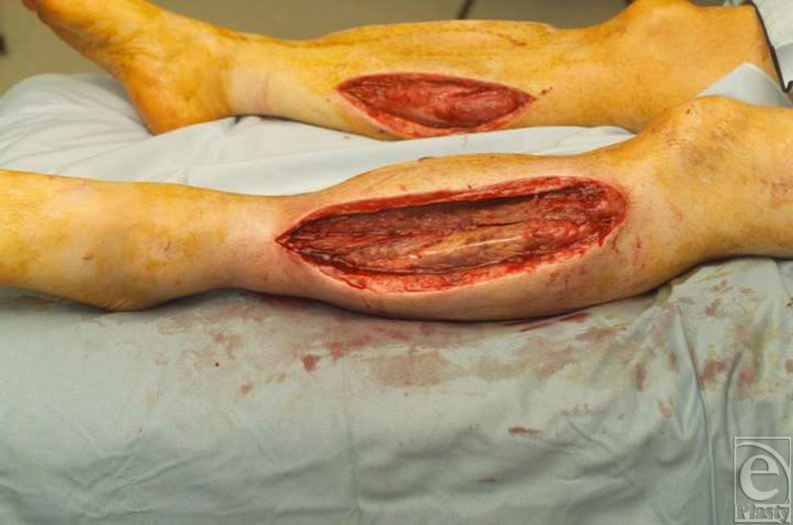
Fasciotomy wound post debridement in both legs.

**Figure 2:**
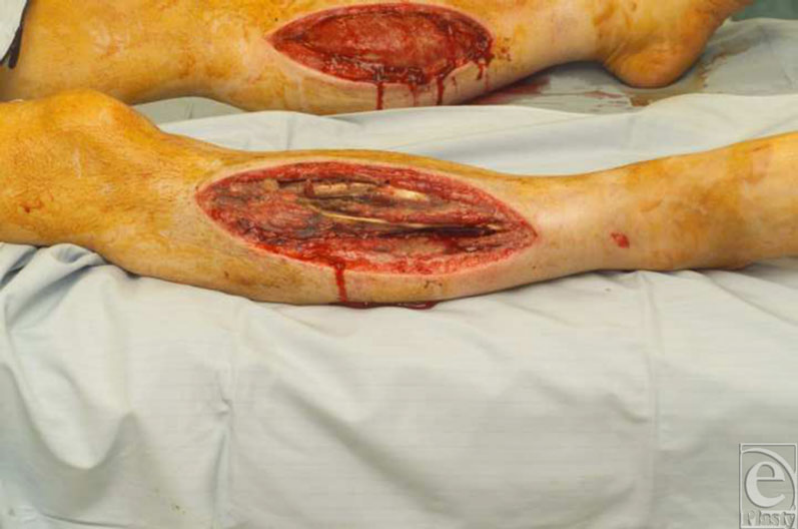
Fasciotomy wound post debridement in both legs.

**Figure 3:**
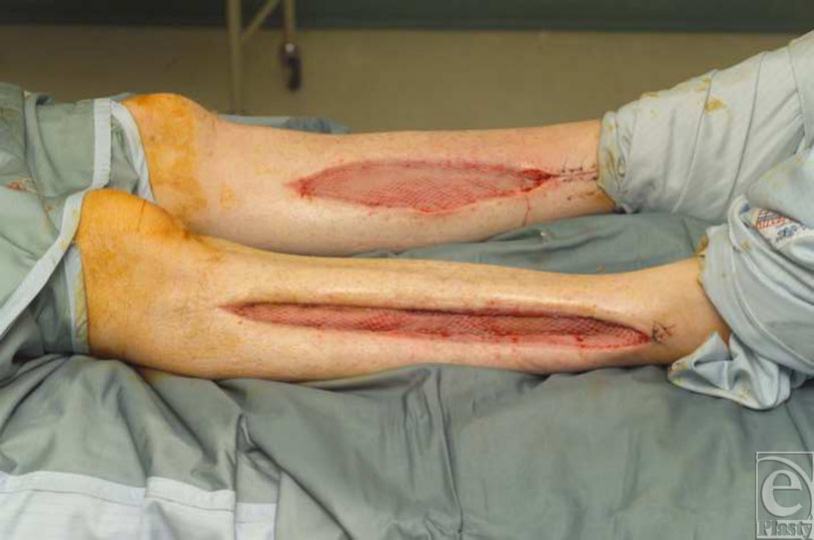
Wounds in both legs post split skin graft.

The patient was later transferred to the plastic surgery unit for the management of fasciotomy wounds. On examination postfasciotomy, the patient had sensory deficit in the distribution of superficial and deep peroneal nerves bilaterally. She had reduced power on plantar flexion and was unable to dorsiflex and evert both feet. The patient was taken to the operation theatre for debridement and exploration of fasciotomy wounds (Figs 1 and 2). In total, 7 debridements were carried out because the patient had progressive necrosis of her peroneal and anterior compartments, as well as necrosis of the left deep compartment involving the tibialis posterior muscle on the left. The right medial longitudinal wound was closed directly. VAC dressings were used for the other wound sites before they were closed with split-thickness skin graft proximally and directly on the distal part (Fig 3). The wound on the lateral left leg could not be primarily closed and a long length of fibula and posterolateral aspect of the tibia was exposed. Free flap surgery was not an option because this was the leg in which there had been a deep vein thrombosis. Over a period of 4 weeks, wound contraction and granulation tissue allowed complete coverage of the fibula. The posterolateral surface of the tibia was covered with skin and a layer of granulation tissue. After split-thickness skin graft was applied, the VAC dressing was reapplied for 5 days. The patient is now 2 months postdiagnosis and is mobilizing with the aid of 2 crutches. She requires orthotic splints bilaterally to correct her foot drop.

## DISCUSSION

Compartment syndrome is a well-known complication posttrauma in the legs. It is, however, rare to develop compartment syndrome after surgery. The overall incidence of well leg compartment syndrome in patients after major pelvic surgery in lithotomy position is estimated at 1 in 3500 cases.^2^ Factors that increase the patient's risk of developing well leg compartment syndrome include procedure duration (>4 hours), lithotomy positioning, Trendelenburg position, ankle dorsiflexion, muscular lower limbs, leg holder type, intermittent pneumatic calf compressors, circumferential wrappings, intraoperative hypotension, hypovolemia, vasoconstrictive drugs, epidural anesthesia, peripheral vascular disease, and surgical retraction on major vessels intraoperatively.^1^ Patients who will be undergoing prolonged surgery in lithotomy position should be screened for these risk factors and adjustments should be made to reduce the risk of developing this complication.

Diagnosis of compartment syndrome is usually made clinically with patient complaining of paresthesia, pain out of proportion to the injury, pain on passive stretch, and tight calves.^3^,^4^ In advanced stages, peripheral pulses are absent. The diagnosis is more difficult in unconscious and sedated patients. Epidural analgesia may have reduced the symptoms of compartment syndrome in this case. This has been reported as a contributing cause for delay in diagnosis of well leg compartment syndrome in several other cases.^2^ However, early diagnosis of compartment syndrome has also been made in patients despite having epidural anesthesia.^5^,^6^ Epidural anesthesia is an effective method of pain control and may be the best option in some patients. Therefore, clinicians' awareness of their patients' risk factors and a high index of suspicion are important during patient assessment.

Compartment pressure can be measured with different pressure catheters. These catheters have fine bore and their use is associated with minimal morbidity.^1^ It is estimated that there are less than 50% of hospitals in the United Kingdom with dedicated measuring devices for compartmental pressure.^4^ This modality may be of benefit if there is any doubt on the diagnosis or in high-risk patients who are sedated or unconscious. Measurements of creatine kinase levels in these patients may also aid diagnosis of well leg compartment syndrome.^1^

It is uncommon for deep vein thrombosis to cause acute compartment syndrome. Massive iliofemoral thrombosis causing compartment syndrome is noted with phlegmasia cerulea dolens. Distal venous obstruction is an unusual cause of compartment syndrome.^7^ Our patient had a deep vein thrombosis in her left leg diagnosed on a Doppler ultrasound scan and compartment syndrome in both lower limbs.

The treatment of compartment syndrome is surgical decompression of the compartments immediately. Fasciotomy is carried out to release the pressure in all 4 compartments (anterior, lateral, superficial posterior, and deep posterior) in the leg. It is important to recognize and treat compartment syndrome early because delayed treatment in patients leads to increased morbidity and mortality.^4^,^8^

The common options for the closure of fasciotomy wounds include split-thickness skin graft and direct closure. Meshed split-thickness skin graft secured with foam vacuum suction dressing after excising all devitalized tissues has been recommended.^4^

The cause of well leg compartment syndrome is multifactorial. It is important that we recognize these risks and carry out appropriate steps to prevent patients from developing it. Raza et al^1^ suggested that patients should be placed in the lithotomy position with minimal elevation of the ankles above the heart level, avoiding head down/Trendelenburg tilt, to remove leg from support every 2 hours for short periods if operating for more than 4 hours, and avoiding ankle dorsiflexion, hypotension, hypovolemia, and vasoconstrictive drugs. They also suggested that intermittent pneumatic compression devices and TED stockings should be avoided in procedures lasting more than 4 hours. Zappa and Sugarbaker^9^ used shoulder braces to prevent movement of their patients on the operating table during steep Trendelenburg position and found that it may play a part in reducing the risk of developing compartment syndrome.

## CONCLUSION

Well leg compartment syndrome is an acute condition associated with significant morbidity and mortality.^4^,^8^ It is a rare but serious complication of surgery. There have been suggestions of ways to prevent this complication. However, more studies looking at the risks, benefits, and feasibility of these suggestions need to be carried before guidelines can be produced for procedures using Lloyd Davies positioning for many hours. Risk factors for developing well leg compartment syndrome include procedure duration (>4 hours), lithotomy positioning, Trendelenburg position with ankle height above the heart level, ankle dorsiflexion, muscular lower limbs, for example, leg holder type, intermittent pneumatic calf compressors, circumferential wrappings, intraoperative hypotension, hypovolemia, vasoconstrictive drugs, epidural anesthesia, peripheral vascular disease, and surgical retraction on major vessels intraoperatively.^1^ Clinicians working with patients who may be at a risk of developing this syndrome need to be aware of its risk factors and have a high index of suspicion when assessing them. Signs and symptoms of severe leg pain, pain to passive stretch, paresthesia, and absent peripheral pulses should make surgeons consider this diagnosis and carry out appropriate measures. Timing of diagnosis and treatment of well leg compartment syndrome is very important because late recognition has a higher risk of serious morbidity and mortality.

## References

[1] Raza A, Byrne D, Townell N (2004). Lower limb (well leg) compartment syndrome after urological pelvic surgery. J Urol.

[2] Simms MS, Terry TR (2005). Well leg compartment syndrome after pelvic ad perineal surgery in the lithotomy position. Postgrad Med J.

[3] Heemskerk J, Kitslaar P. (2003). Acute compartment syndrome of the lower leg: retrospective study on prevalence, technique and outcome of fasciotomies. World J Surg.

[4] Pearse M, Harry L, Nanchahal J (2002). Acute compartment syndrome of the leg. BMJ.

[5] Beerle BJ, Rose RJ (1993). Lower extremity compartment syndrome from prolonged lithotomy position not masked by epidural bupivacaine and fentanyl. Reg Anesth.

[6] Montgomery CJ, Ready LB (1991). Epidural opioid analgesia does not obscure diagnosis of compartment syndrome resulting from prolonged lithotomy position. Anaesthesiology.

[7] Vanfleet TA, Raad MG, Watson MD (1996). Popliteal vein thrombosis causing compartment syndrome: a case report. Clin Orthopaed Relat Res.

[8] Finkelstein JA, Hunter GA, Hu RW (1996). Lower limb compartment syndrome: course after delayed fasciotomy. J Trauma.

[9] Zappa L, Sugarbaker PH (2007). Compartment syndrome of the leg associated with lithotomy position for cytoreductive surgery. J Surg Oncol.

